# Fondaparinux sodium and low molecular weight heparin for venous thromboembolism prophylaxis in Chinese patients with major orthopedic surgery or trauma: a real-world study

**DOI:** 10.1186/s12893-022-01652-6

**Published:** 2022-06-24

**Authors:** Donglin Fu, Li Li, Yifan Li, Xiaofei Liu, Hongkang Chen, Naitian Wu, Guangfeng Sun

**Affiliations:** Joint and Trauma Ward, Department of Orthopaedics, Fuyang People’s Hospital, No. 501 Sanqing Road, Yingzhou District, Fuyang, 236000 Anhui Province China

**Keywords:** Fondaparinux sodium, Low molecular weight heparin, Venous thromboembolism prophylaxis, Major orthopedic surgery, Trauma

## Abstract

**Background:**

The present real-world study aimed to compare the efficacy and safety between fondaparinux sodium (FPX) and low molecular weight heparin (LMWH) for venous thromboembolism (VTE) prophylaxis in Chinese patients with major orthopedic surgery or trauma.

**Methods:**

A total of 2429 patients, with major orthopedic surgery or trauma, underwent FPX (n = 1177) or LMWH (n = 1252) for VTE prophylaxis and were retrospectively reviewed. Primary outcomes, including in-hospital VTE and in-hospital major bleeding incidences, as well as the secondary outcomes, including in-hospital minor bleeding, in-hospital death, and VTE/bleeding/death within 2 months after discharge, were analyzed. Inverse probability of treatment weighting (IPTW) was conducted.

**Results:**

FPX group exhibited lower in-hospital VTE (0.1% vs. 0.8%; *P* = 0.032, crude OR = 0.11 before IPTW; *P* = 0.046, weighted OR = 0.12 after IPTW) and in-hospital minor bleeding (17.8% vs. 26.8%; *P* < 0.001, crude OR = 0.59 before IPTW; *P* < 0.001, weighted OR = 0.67 after IPTW) compared to LMWH group. Furthermore, no difference of in-hospital major bleeding, in-hospital death, and VTE/bleeding/death within 2 months after discharge was observed between FPX group and LMWH group (all *P* > 0.05). Further subgroup analyses identified, in specific cluster of patients such as older age, renal function impairment, hypertension and so on, in-hospital VTE was declined in FPX group compared to LMWH group (all *P* < 0.001).

**Conclusions:**

FPX is probable to exhibit a superior thromboprophylaxis efficacy compared with LMWH in in-hospital patients with major orthopedic surgery or trauma, especially in some special patients such as older age, renal function impairment, hypertension, etc.

## Background

Venous thromboembolism (VTE), consisting of deep vein thrombosis (DVT) and pulmonary embolism (PE), attacks approximately ten million populations annually over the world [1]. Multiple provoking risk factors are considered for the VTE development, such as major surgery, active cancer, major trauma or fracture, antiphospholipid syndrome and so on [2]. Among the above conditions, patients receiving major orthopedic surgery (including total knee arthroplasty (TKA), total hip arthroplasty (THA), hip fractures surgery (HFS), etc.) or experiencing severe trauma are at high risk of VTE [3–5]. Moreover, those patients occurring VTE not only suffer from clinical symptoms like leg pain, swelling and localized tenderness, bare high-stake disability and mortality, but also endure health-care economic burdens [6–9]. Therefore, the efforts to prevent VTE for major orthopedic surgery or trauma have never been stopped [1].

VTE prophylaxis is presently recommended for major orthopedic surgery or trauma by several guidelines such as American College of Chest Physicians (ACCP) guideline and Chinese Orthopaedic Association guideline, with use of unfractioned heparin (UFH), low molecular weight heparin (LMWH), factor Xa inhibitor, vitamin K antagonist (VKA) or antiplatelet drug after the consideration of disease conditions [10, 11]. Fondaparinux sodium (FPX), as a classic factor Xa inhibitor, exhibits liner thrombin-inhibiting effect with bottleneck constraint and does not bind to platelet Factor 4, which reduces the risk of over anticoagulation and thrombocytopenia [12, 13]. Several randomized, controlled trials have presented the superiority of FPX over LMWH for VTE prophylaxis in patients underwent major orthopedic surgery [14–17]. However, data in aspect to FPX for VTE prophylaxis, under real-clinical settings and its effect in Chinese patients with major orthopedic surgery or trauma, is still lacking.

Thus, the current real-world study aimed to compare the efficacy and safety between FPX and LMWH for VTE prophylaxis in Chinese patients with major orthopedic surgery or trauma.

## Methods

### Study population

This was a single-center, retrospective, cohort study based on clinical data collected from real-world medical conditions in China. The patients who used FPX or LMWH for the prevention of VTE for patients suffering from major orthopedic surgery (including hip fracture, hip replacement, knee replacement, and upper limb surgery) and trauma (including the traffic accident, brawl, occupational injury, and high-altitude falling) in the Fuyang People's Hospital between December 14, 2016 and August 25, 2020 were included in the study. The screening criteria for patients were as follows: (1) trauma patients or patients undergoing major orthopedic surgery, with the use of FPX or LMWH for the prevention of VTE; (2) had complete clinical information and follow-up data. The exclusion criteria included: (1) used other anticoagulants apart from FPX or LMWH during the hospitalization; (2) underwent ≥ 2 times of total hip replacement (THR) or total knee replacement (TKR) during the study period; (3) diagnosed as VTE at admission. A total of 2429 patients meeting the enrollment criteria were included in the study, and there were 1177 patients who received FPX treatment in the FPX group and 1252 patients who received LMWH treatment in the LMWH group, resulting in an enrollment ratio about 1:1 between two groups. This study was conducted according to Declaration of Helsinki, and was approved by Institutional Review Board of the hospital. Due to the non-interventional, retrospective design, no sample requirement, only AE regarding VTE data was reviewed, the informed consents were waivered approved by Institutional Review Board of Fuyang People’s Hospital.

### Collection of clinical data

Based on the research protocol, clinical data were collected from the hospital database, including (1) clinical features: demographic characteristics, risk factors, DVT score, vital signs, and laboratory indexes; (2) treatment information: types of surgery, types of anesthesia duration of surgery, duration of anesthesia, duration of medication and hospital length of stay (HLOS); (3) events occurred in hospital and within 2 months after discharge: VTE, bleeding, and death. All data were anonymized to protect patient privacy.

### Administration of FPX and LMWH

FPX (Hengrui Medicine Co., Ltd, Lianyungang, Jiangsu, China) and LMWH (without restrictions of manufacturers and types) were all administered by subcutaneous injection for the prevention of VTE. In details, in general, the FPX was applied as follows: FPX, 2.5 mg per day, subcutaneous injection for 14 days beginning 24 h after discontinuation of anesthesia; The LMWH was applied as follows: LMWH, 4000–4100 UI per day, subcutaneous injection for 14 days beginning 24 h after discontinuation of anesthesia.

### Assessment of outcomes

The primary outcomes were symptomatic VTE in hospital and major bleeding in hospital. The secondary outcomes included minor bleeding in hospital, death in hospital, VTE within 2 months after discharge, bleeding within 2 months after discharge, and death within 2 months after discharge. The time for the assessment of primary-endpoint VTE was on the day of discharge. Besides, the time for the assessment of secondary-endpoint VTE was on the day of 2nd month after discharge or the occurrence of patients’ death.

### Definitions

The VTE included symptomatic PE, symptomatic DVT and asymptomatic DVT. Major bleeding included fatal bleeding, significant bleeding (bleeding causing a drop in hemoglobin level of 20 g/L or more, or leading to transfusion of two or more units of whole blood or red cells), bleeding at critical position (significant bleeding in a critical area or organ, such as intracranial, intraspinal, intraocular, retroperitoneal, intraarticular or pericardial, or intramuscular with compartment syndrome). Minor bleeding included clinically relevant non-major bleeding (the significant bleeding which does not met the criteria for major bleeding, but required medical intervention) and other minor bleedings.

### Statistical analysis

Under the missing at random assumption, the missing data were imputed using mean for height (0.1% missing), platelet (PLT) (4.9%), thrombin time (TT) (0.5%), D-dimer (0.5%), prothrombin time activity (PTA) (0.4%), fibrinogen (FIB) (7.2%) and creatinine clearance rate (Ccr) (4.3%). We compared the baseline characteristics between patients who received FPX and LMWH. SMD approach was used to evaluate the balance in covariates (SMD < 0.1 was considered as negligible imbalance between groups). Considering the confounding bias against basic clinical features, inverse probability of treatment weighting (IPTW) method was applied to balance the differences in clinical features between 2 intervention groups. To be specifical, the propensity score (PS), the conditional probability of receiving FPX, was estimated using a multivariate logistic regression model based on the factors including height, body mass indexes (BMI), PLT, prothrombin time (PT), D-dimer, PTA, FIB, Ccr, type of surgery (hip fracture, hip replacement, knee replacement, upper limb surgery, other trauma surgery and with no surgery), type of anesthesia (general anesthesia and regional anesthesia) and duration of surgery. PS was then used to weight each patient between the 2 groups. Logistic regression models were applied to estimate the association between primary outcomes and groups on IPTW dataset and subgroups analysis. R 4.0.2 software packages (R Core Team 2021) were used for statistical analyses. All *P* values were two sided. A *P* value of < 0.05 was considered statistically significant.

## Results

### Patients’ characteristics

**Fig. 1:**
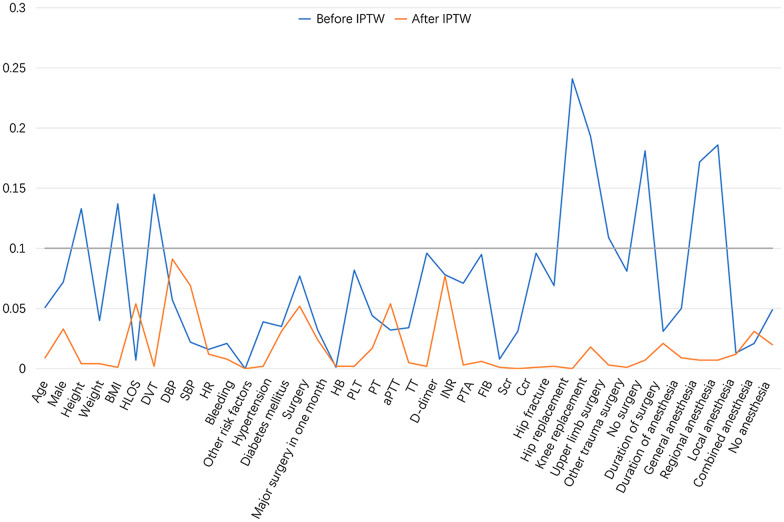
Balance of parameters between two groups after inverse-probability-of-treatment weighting method (IPTW)

The mean age was 60.3 ± 16.4 years in FPX group while 61.1 ± 14.5 years in LMWH group (SMD = 0.051) before IPTW, then was 60.9 ± 15.8 years in FPX group while 60.8 ± 15.0 years in LMWH group (SMD = 0.009) after IPTW. The proportion of males was 43.3% in FPX group while 39.8% in LMWH group (SMD = 0.072) before IPTW, then was 40.5% in FPX group while 42.2% in LMWH group (SMD = 0.033) after IPTW. The detailed information about other clinical characteristics, in aspect to risk factors, vital signs, laboratory indexes, etc., between the two groups is exhibited in Table 1. In addition, patients’ characteristics were balanced between the two group after IPTW, the balance was much improved compared to that before IPTW (Fig. 1, Table 1).

**Table 1:** Clinical characteristics

Items	Before IPTW			After IPTW		
	FPX (N = 1177)	LMWH (N = 1252)	SMD	FPX	LMWH	SMD
Demographic characteristics						
Age (years), mean ± SD	60.3 ± 16.4	61.1 ± 14.5	0.051	60.9 ± 15.8	60.8 ± 15.0	0.009
Male, No. (%)	510 (43.3)	498 (39.8)	0.072	40.5	42.2	0.033
Height (cm), mean ± SD	163.8 ± 8.3	162.7 ± 8.6	0.133	163.2 ± 8.3	163.3 ± 8.7	0.004
Weight (Kg), mean ± SD	65.2 ± 11.4	65.7 ± 11.7	0.040	65.4 ± 11.4	65.5 ± 11.9	0.004
BMI (Kg/m^2^), mean ± SD	24.3 ± 3.7	24.8 ± 3.9	0.137	24.6 ± 3.8	24.6 ± 3.9	0.001
Risk factors, No. (%)						
History of bleeding	21 (1.8)	19 (1.5)	0.021	1.7	1.8	0.008
History of hypertension	402 (34.2)	451 (36.0)	0.039	35.4	35.3	0.002
History of diabetes mellitus	93 (7.9)	111 (8.9)	0.035	8.1	9.0	0.031
History of surgery	292 (24.8)	353 (28.2)	0.077	25.2	27.5	0.052
Major surgery within one month	5 (0.4)	3 (0.2)	0.032	0.4	0.3	0.024
Other risk factors	1177 (100.0)	1252 (100.0)	< 0.001	100.0	100.0	< 0.001
DVT score, mean ± SD	10.2 ± 4.1	9.7 ± 3.9	0.145	10.0 ± 3.9	10.0 ± 4.1	0.002
Vital signs, mean ± SD						
DBP (mmHg)	78.6 ± 11.2	78.0 ± 10.7	0.057	78.8 ± 11.3	77.8 ± 10.8	0.091
SBP (mmHg)	136.0 ± 17.8	135.6 ± 17.0	0.022	136.4 ± 17.7	135.2 ± 17.1	0.069
HR (times/min)	79.4 ± 8.9	79.2 ± 8.8	0.016	79.3 ± 8.8	79.4 ± 9.0	0.012
Laboratory indexes, mean ± SD						
HB (g/L)	128.4 ± 15.6	128.5 ± 14.9	0.001	128.5 ± 15.4	128.5 ± 15.2	0.002
PLT (X10^9^/L)	233.1 ± 71.6	239.1 ± 74.1	0.082	236.3 ± 73.4	236.4 ± 72.7	0.002
PT (s)	12.8 ± 5.2	12.6 ± 5.4	0.044	12.8 ± 5.5	12.9 ± 8.1	0.017
aPTT (s)	30.5 ± 7.1	30.3 ± 8.4	0.032	30.8 ± 7.6	30.3 ± 8.1	0.054
TT (s)	18.7 ± 8.0	18.5 ± 2.5	0.034	18.6 ± 7.0	18.5 ± 2.5	0.005
D-dimer (mg/L)	5.8 ± 12.4	4.6 ± 11.7	0.096	5.2 ± 11.8	5.1 ± 12.1	0.002
INR	1.1 ± 0.9	1.2 ± 3.1	0.078	1.1 ± 1.0	1.2 ± 3.0	0.077
PTA (%)	106.1 ± 19.3	108.3 ± 37.7	0.071	106.7 ± 19.4	106.8 ± 33.6	0.003
FIB (g/L)	2.9 ± 0.8	3.0 ± 0.8	0.095	3.0 ± 0.8	3.0 ± 0.8	0.006
Scr (μmol/L)	71.2 ± 40.2	70.8 ± 45.9	0.008	71.0 ± 39.9	71.0 ± 46.0	0.001
Ccr (mL/min)	102.5 ± 38.6	101.4 ± 36.6	0.031	101.8 ± 37.6	101.8 ± 37.1	< 0.001
Type of surgery, No. (%)						
Hip fracture	186 (15.8)	156 (12.5)	0.096	14.1	14.0	0.001
Hip replacement	160 (13.6)	201 (16.1)	0.069	14.8	14.8	0.002
Knee replacement	296 (25.1)	453 (36.2)	0.241	30.8	30.8	< 0.001
Upper limb surgery	37 (3.1)	7 (0.6)	0.193	1.8	1.6	0.018
Other trauma surgery	465 (39.5)	429 (34.3)	0.109	37.0	37.1	0.003
No surgery	72 (6.1)	54 (4.3)	0.081	5.2	5.3	0.001
Type of anesthesia, No. (%)						
General anesthesia	357 (30.3)	285 (22.8)	0.172	26.3	26.0	0.007
Regional anesthesia	691 (58.7)	847 (67.7)	0.186	63.4	63.7	0.007
Local anesthesia	17 (1.4)	20 (1.6)	0.013	1.6	1.4	0.012
Combined anesthesia	18 (1.5)	16 (1.3)	0.021	1.6	1.3	0.031
No anesthesia	94 (8.0)	84 (6.7)	0.049	7.1	7.6	0.020
Duration of surgery (hours), mean ± SD	1.9 ± 1.1	1.8 ± 0.9	0.181	1.8 ± 1.0	1.9 ± 1.1	0.007
Duration of anesthesia (hours), mean ± SD	3.6 ± 24.5	3.0 ± 19.5	0.031	3.4 ± 22.7	4.0 ± 28.7	0.021
Duration of medication (days), mean ± SD	8.1 ± 12.2	7.5 ± 11.5	0.050	7.7 ± 11.7	7.8 ± 12.7	0.009
HLOS (days), mean ± SD	10.9 ± 6.4	10.9 ± 5.6	0.007	10.7 ± 5.9	11.0 ± 6.2	0.054

*IPTW* inverse-probability-of-treatment weighting method, *FPX* fondaparinux sodium, *LMWH* low molecular weight heparins, *SMD* standardized mean difference, *SD* standard deviation, *BMI* body mass indexes, *DVT* deep vein thrombosis, *DBP* diastolic blood pressure, *SBP* systolic blood pressure, *HR* heart rate, *HB* hemoglobin, *PLT* platelet, *PT* prothrombin time, *aPTT* activated partial thromboplastin time, *TT* thrombin time, *INR* international normalized ratio, *PTA* prothrombin time activity, *FIB* fibrinogen *Scr* serum creatinine, *Ccr* creatinine clearance rate, *HLOS* hospital length of stay

### Outcomes

**Fig. 2:**
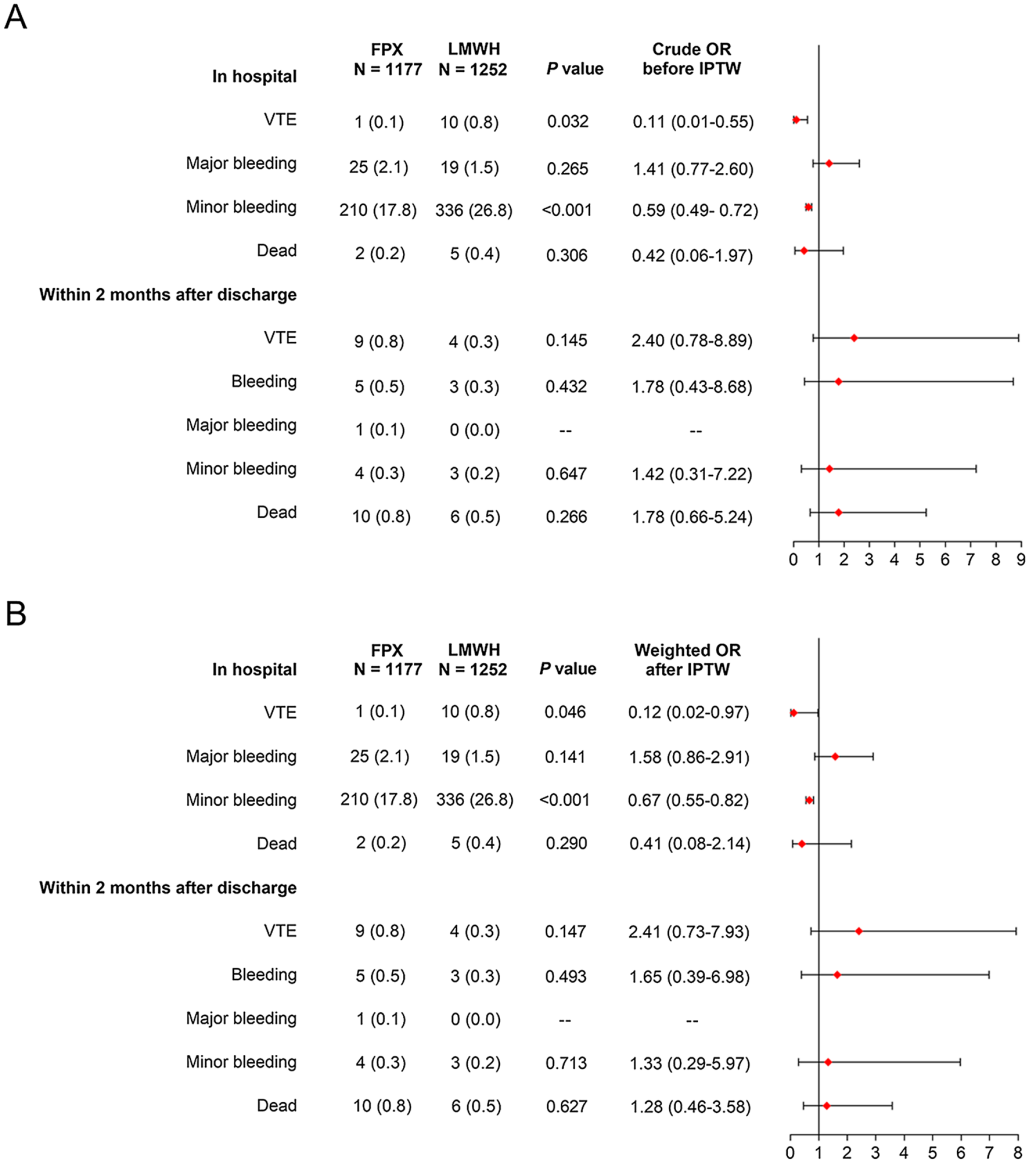
Outcome assessments. Comparison of outcomes between FPX group and LMWH group before IPTW (**A**) and after IPTW (**B**). IPTW, inverse-probability-of-treatment weighting method

Before IPTW: FPX group exhibited lower in-hospital VTE (0.1% vs. 0.8%, *P* = 0.032, crude OR = 0.11) and in-hospital minor bleeding (17.8% vs. 26.8%, *P* < 0.001, crude OR = 0.59), but similar in-hospital major bleeding and in-hospital death (both *P* > 0.05), compared to LMWH group (Fig. 2A); Furthermore, no difference of VTE, total bleeding, major bleeding, minor bleeding and death within 2 months after discharge was observed between FPX group and LMWH group (all *P* > 0.05).

After IPTW: FPX group also showed decreased in-hospital VTE (0.1% vs. 0.8%, *P* = 0.046, weighted OR = 0.12) and in-hospital minor bleeding (17.8% vs. 26.8%, *P* < 0.001, weighted OR = 0.67), while equal in-hospital major bleeding and in-hospital death (both *P* > 0.05), compared with LMWH group (Fig. 2B); In addition, VTE, total bleeding, major bleeding, minor bleeding and death within 2 months after discharge were of no difference between FPX group and LMWH group (all *P* > 0.05).

### Subgroup analyses

Further comparison of in-hospital VTE and in-hospital major bleeding between FPX administration and LMWH administration in subgroups was also conducted. In-hospital VTE after IPTW was declined by FPX administration compared to LMWH administration in patients with older age (*P* < 0.001), patients with light to moderate impairment of renal function (*P* < 0.001), patients with hypertension (*P* < 0.001), patients with other trauma surgery (*P* < 0.001), patients with premedicate time > 0 h and > 24 h (*P* < 0.001), patients with 0–5 days of drug administration (*P* < 0.001), patients with 10–15 days of drug administration (*P* < 0.001), patients with both major orthopedic surgery and moderate impairment of renal function (*P* < 0.001) (Table 2); but was of no difference between the two administrations in other subgroups (all *P* > 0.05).

**Table 2:** Subgroup analysis of VTE in hospital (after IPTW)

Items	FPX (N = 1177)	LMWH (N = 1252)	IPTW-weighted OR	*P* value
	n/N (%)	n/N (%)		
Age				
18–59 years	1/528 (0.2)	4/500 (0.8)	0.32 (0.04–2.89)	0.311
≥ 60 years	0/649 (0.0)	6/752 (0.8)	–	< 0.001
60–79 years	0/518 (0.0)	6/656 (0.9)	–	< 0.001
≥ 80 years	0/131 (0.0)	0/96 (0.0)	–	–
Weight ≤ 50 kg	0/125 (0.0)	0/135 (0.0)	–	–
Impairment of renal function				
Normal	1/541 (0.2)	5/469 (1.1)	0.23 (0.01–1.43)	0.179
Light	0/242 (0.0)	1/261 (0.4)	–	< 0.001
Moderate	0/75 (0.0)	1/68 (1.5)	–	< 0.001
Complication				
Diabetes mellitus	0/93 (0.0)	0/111 (0.0)	–	–
Hypertension	0/402 (0.0)	4/451 (0.9)	–	< 0.001
Tumor	0/35 (0.0)	0/32 (0.0)	–	–
Anemia	0/69 (0.0)	0/49 (0.0)	–	–
Different types of surgery^a^				
Major orthopedic surgery	1/625 (0.2)	8/780 (1.0)	0.19 (0.02–1.54)	0.121
Other trauma surgery	0/500 (0.0)	2/436 (0.5)	–	< 0.001
No surgery	0/72 (0.0)	0/54 (0.0)	–	–
Different types of surgery^b^				
Hip and knee replacement	1/456 (0.2)	8/654 (1.2)	0.22 (0.03–1.74)	0.150
Trauma surgery	0/673 (0.0)	2/583 (0.3)	–	< 0.001
No surgery	0/72 (0.0)	0/54 (0.0)	–	–
Premedicate				
> 0 h	0/476 (0.0)	1/337 (0.3)	–	< 0.001
> 24 h	0/450 (0.0)	1/308 (0.3)	–	< 0.001
Different days of administration				
0–5 days	0/636 (0.0)	2/630 (0.3)	–	< 0.001
6–9 days	1/376 (0.3)	7/450 (1.6)	0.17 (0.02–1.44)	0.106
10–15 days	0/120 (0.0)	1/132 (0.8)	–	< 0.001
16–35 days	0/41 (0.0)	0/37 (0.0)	–	–
> 35 days	0/4 (0.0)	0/2 (0.0)	–	–
Duration of surgery				
≤ 45 min	0/40 (0.0)	0/32 (0.0)	–	–
> 45 min	1/1029 (0.1)	9/1092 (0.8)	0.14 (0.02–1.11)	0.063
Surgery and impairment of renal function				
Major orthopedic surgery and moderate	0/43 (0.0)	1/48 (2.1)	–	< 0.001
Other trauma surgery and moderate	0/25 (0.0)	0/13 (0.0)	–	–

^a^Major orthopedic surgery: hip replacement, knee replacement and hip fracture; Other trauma surgery: upper limb surgery and other trauma surgery. ^b^Hip and knee replacement: hip replacement and knee replacement; Trauma surgery: hip fracture; upper limb surgery and other trauma surgery. Impairment of renal function was classified as normal (Ccr ≥ 90 mL/min), light (Ccr: 60–89 mL/min), moderate (Ccr: 30–59 mL/min). *VTE* venous thromboembolism, *IPTW* inverse-probability-of-treatment weighting method, *FPX* fondaparinux sodium, *LMWH* low molecular weight heparins, *OR* odds ratio

In-hospital major bleeding after IPTW was similar between FPX administration and LMWH administration in the most of subgroups (all *P* < 0.05) (Table 3). However, it was decreased by FPX administration compared to LMWH administration in patients with tumor (*P* < 0.001); oppositely, it was increased by FPX administration compared to LMWH administration to some extent in patients with light impairment of renal function (*P* = 0.010), patients with moderate impairment of renal function (*P* < 0.001), patients with both major orthopedic surgery and moderate impairment of renal function (*P* < 0.001), and patients with other trauma surgery and moderate impairment of renal function (*P* < 0.001).

**Table 3:** Subgroup analysis of major bleeding in hospital (after IPTW)

Items	FPX (N = 1177)	LMWH (N = 1252)	IPTW-weighted OR	*P* value
	n/N (%)	n/N (%)		
Age				
18–59 years	8/528 (1.5)	7/500 (1.4)	1.61 (0.57–4.61)	0.371
≥ 60 years	17/649 (2.6)	12/752 (1.6)	1.57 (0.74–3.34)	0.244
60–79 years	9/518 (1.7)	10/656 (1.5)	1.12 (0.44–2.80)	0.815
≥ 80 years	8/131 (6.1)	2/96 (2.1)	2.52 (0.52–12.2)	0.254
Weight ≤ 50 kg	6/125 (4.8)	1/135 (0.7)	5.16 (0.60–44.19)	0.135
Impairment of renal function				
Light	9/242 (3.7)	1/261 (0.4)	15.75 (1.97–126.14)	0.010
Moderate	5/75 (6.7)	0/68 (0.0)	–	< 0.001
Normal	7/541 (1.3)	14/469 (3.0)	0.57 (0.21–1.38)	0.229
Complication				
Diabetes mellitus	4/93 (4.3)	3/111 (2.7)	2.66 (0.56–12.67)	0.222
Hypertension	8/402 (2.0)	7/451 (1.6)	1.65 (0.58–4.68)	0.350
Tumor	0/35 (0.0)	1/32 (3.1)	–	< 0.001
Anemia	7/69 (10.1)	2/49 (4.1)	4.44 (0.84–23.41)	0.081
Different types of surgery^a^				
Major orthopedic surgery	20/625 (3.2)	18/780 (2.3)	1.46 (0.75–2.83)	0.267
Other trauma surgery	5/500 (1.0)	1/436 (0.2)	4.31 (0.50–37.32)	0.185
No surgery	0/72 (0.0)	0/54 (0.0)	–	–
Different types of surgery^b^				
Hip and knee replacement	16/456 (3.5)	15/654 (2.3)	1.57 (0.76–3.24)	0.225
Trauma surgery	10/673 (1.5)	4/583 (0.7)	2.09 (0.65–6.72)	0.218
No surgery	0/72 (0.0)	0/54 (0.0)	–	–
Premedicate				
> 0 h	11/476 (2.3)	5/337 (1.5)	1.69 (0.57–4.99)	0.343
> 24 h	11/450 (2.4)	4/308 (1.3)	2.16 (0.66–7.06)	0.201
Different days of administration				
0–5 days	7/636 (1.1)	7/630 (1.1)	0.95 (0.33–2.76)	0.921
6–9 days	16/376 (4.3)	9/450 (2.0)	2.35 (1.00–5.49)	0.050
10–15 days	1/120 (0.8)	2/132 (1.5)	3.39 (0.30–38.69)	0.326
16–35 days	1/41 (2.4)	1/37 (2.7)	0.57 (0.03–9.75)	0.696
> 35 days	0/4 (0.0)	0/2 (0.0)	–	–
Duration of surgery				
≤ 45 min	0/40 (0.0)	0/32 (0.0)	–	–
–	25/1029 (2.4)	17/1092 (1.6)	1.75 (0.93–3.29)	0.082
Surgery and impairment of renal function				
Major orthopedic surgery and moderate	2/43 (4.7)	0/48 (0.0)	–	< 0.001
Other trauma surgery and moderate	3/25 (12.0)	0/13 (0.0)	–	< 0.001

^a^Major orthopedic surgery: hip replacement, knee replacement and hip fracture; Other trauma surgery: upper limb surgery and other trauma surgery. ^b^Hip and knee replacement: hip replacement and knee replacement; Trauma surgery: hip fracture; upper limb surgery and other trauma surgery. *IPTW* inverse-probability-of-treatment weighting method, *FPX* fondaparinux sodium, *LMWH* low molecular weight heparins, *OR* odds ratio

## Discussion

This was the first real-world study focusing on FPX for VTE prophylaxis in Chinese patients with major orthopedic surgery or trauma, which uncovered several interesting findings as follows: (1) FPX realized lower in-hospital VTE and in-hospital minor bleeding compared to LMWH; (2) FPX exhibited similar in-hospital major bleeding and in-hospital death, as well as equal VTE, total bleeding, major bleeding, minor bleeding and death within 2 months after discharge compared to LMWH; (3) subgroup analyses further identified several subgroup populations in which FPX showed better efficacy than LMWH for preventing VTE.

VTE is initially a deadly complication engaged in the major orthopedic surgery or trauma, since the introduction of efficient prophylaxis method, its incidence and related mortality are greatly declined, therefore the VTE prophylaxis is commonly recommended in these patients [1, 11]. In detail, the recent Chinese Orthopaedic Association guideline reports the incidence of DVT ranging from 0.26 to 6.00% in Europe and America, ranging from 0.20 to 3.50% in Asia, and specifically ranging from 2.40 to 16.10% in China; it also reveals the incidence of PE ranging from 0.14 to 4.60% in Europe and America, ranging from 0.00 to 2.40% in Asia, and particularly ranging from 0.00 to 0.47% in China [11]. Currently, the marketed anticoagulants in China mainly include UFH, LMWH, factor Xa inhibitor, VKA, direct oral administration of anticoagulants (DOAC) or antiplatelet drug. However, the narrow therapeutic-window duration of UFH may lead to elevated risk of major bleeding and Heparin induced thrombocytopenia (HIT) risk exists; LMWH although reduces major bleeding occurrence, while it also relates to HIT risk; VKA bears narrow treatment dose and obvious individual variation, leading to routing monitor of international normalized ratio (INR) as necessary to avoid major bleeding, meanwhile, its effect is commonly affected by other drugs and food, and its onset time as well as half-time period are relatively long. DOAC, such as the rivaroxaban and apixaban, etc., is also routinely used in the clinical practice for the thromboprophylaxis. Besides, some studies have shown its benefits over parenteral treatment; for instance, one META-analysis shows that apixaban is associated with a reduction in the risk of major/clinically relevant nonmajor bleeding events compared to LMWH [18].

FPX, as the first chemically synthesized methoxy derivative of the natural pentasaccharide sequence, binds to antithrombin selectively then catalyzes the repression of Factor Xa quickly, which produces a transformation of configuration leading to an around 300-fold increment in the natural inactivation of antithrombin against Factor Xa [19–21]. FPX does not interact with platelets, nor does it affect bleeding time, activate partial thromboplastin time, prothrombin time [20, 21]. Benefiting from the above effects and advantages, FPX has been widely used and commonly recommended for the VTE prophylaxis of major orthopedic surgery or trauma [10, 11]. As for the clinical superiority of FPX, PENTATHALON trial observes that VTE occurrence by day 11 is 6% in cases on FPX while 8% in cases on LMWH with relative reduction in risk of 26.3% in patients underwent elective hip-replacement surgery, meanwhile, their major bleeding and death incidence shows no difference [14]; EPHESUS trial also discovers an obviously lower VTE occurrence with day 11 by FPX compared to LMWH (4% vs. 9%, relative reduction in risk of 55.9%), while similar in death and major bleeding risk in patients with elective hip-replacement surgery [15]; furthermore, PENTAMAKS trial (VTE 12.5% vs. 27.8%, relative reduction risk 55.2%) and PENTHIFRA trial (VTE 8.3% vs. 19.1%, relative reduction risk 56.4%) also demonstrate similar findings that FPX provides more benefits than LMWH for VTE prophylaxis in patients with elective major knee surgery and hip-fracture surgery [16, 17]. However, these previous famous trials are mainly conducted in Europe and America, while the study focusing on FPX in Chinese patients is limited, the data in terms of real-clinical conditions is lacking. Therefore, we performed the current real-world study, which observed that FPX realized lower in-hospital VTE and in-hospital minor bleeding compared to LMWH, also it exhibited similar in-hospital major bleeding and in-hospital death, as well as equal VTE, total bleeding, major bleeding, minor bleeding and death within 2 months after discharge compared to LMWH in Chinses patients with major orthopedic surgery or trauma. The possible explanations were as follows: (1) The superior selective inhibition of factor Xa rapidly, predictable linear pharmacokinetics, and relatively long half-life time contributed to the better VTE prophylaxis of FPX over LMWH [13, 16]; (2) FPX did not interact with platelets nor did it affect bleeding time, activate partial thromboplastin time, prothrombin time, therefore realized a lower minor bleeding and equal major bleeding in the studied patients [20, 21].

Subgroup analyses were performed to further identify the specific clusters of patients in which FPX shows superiority to LMWH in patients with major orthopedic surgery or trauma. Inspiringly, we discovered that in patients with older age, patients with light to moderate impairment of renal function, patients with hypertension, patients with other trauma surgery, patients with premedicate time > 0 h and > 24 h, patients with 0–5 days of drug administration, patients with 10–15 days of drug administration, patients with both major orthopedic surgery and moderate impairment of renal function, FPX was better for VTE prophylaxis compared to LMWH. This finding highlights the specific patient type among which FPX maybe is an optimized choice for VTE prophylaxis.

Although some interesting findings were uncovered in our present study, several limitations should be clarified: (1) this was a single-center study, therefore selection bias was an unavoidable issue; (2) this was a retrospective real-world study, thus some missing data existed which might influence the results, such as the dosages and duration of anti-thrombotic treatment; (3) since the incidence of VTE or major bleeding was relatively low, and the sample size of the study was hard to greatly enlarged, these made some subgroup analyses lacking sufficient statistical power; (4) the fondaparinux might increase the healthcare spending due to its high cost, therefore, a study which evaluated its cost-effectiveness was needed.

## Conclusion

In conclusion, FPX is probable to exhibit a superior thromboprophylaxis efficacy compared with LMWH in in-hospital patients with major orthopedic surgery or trauma, especially in some special patients such as older age, renal function impairment, hypertension, etc.

## Data Availability

All data generated or analysed during this study are included in this published article.

## Abbreviations

VTE: Venous thromboembolism
DVT: Deep vein thrombosis
PE: Pulmonary embolism
TKA: Total knee arthroplasty
THA: Total hip arthroplasty
HFS: Hip fractures surgery
ACCP: American College of Chest Physicians
UFH: Unfractioned heparin
LMWH: Low molecular weight heparin
FPX: Fondaparinux sodium
THR: Total hip replacement
TKR: Total knee replacement
HLOS: Hospital length of stay
PLT: Platelet
TT: Thrombin time
PTA: Prothrombin time activity
FIB: Fibrinogen
Ccr: Creatinine clearance rate
IPTW: Inverse probability of treatment weighting
PS: Propensity score
BMI: Body mass indexes
PT: Prothrombin time
HIT: Heparin induced thrombocytopenia
INR: International normalized ratio
